# Sequential Change in T2* Values of Cartilage, Meniscus, and Subchondral Bone Marrow in a Rat Model of Knee Osteoarthritis

**DOI:** 10.1371/journal.pone.0076658

**Published:** 2013-10-18

**Authors:** Ping-Huei Tsai, Herng-Sheng Lee, Tiing Yee Siow, Yue-Cune Chang, Ming-Chung Chou, Ming-Huang Lin, Chien-Yuan Lin, Hsiao-Wen Chung, Guo-Shu Huang

**Affiliations:** 1 Imaging Research Center, Taipei Medical University, Taipei, Taiwan; 2 Department of Medical Imaging, Taipei Medical University Hospital, Taipei Medical University, Taipei, Taiwan; 3 Department of Pathology, Tri-Service General Hospital, National Defense Medical Center, Taipei, Taiwan; 4 Department of Medical Imaging and Intervention, Chang Gung Memorial Hospital, College of Medicine, Chang Gung University, Taoyuan, Taiwan; 5 Institute of Biomedical Sciences, Academic Sinica, Taipei, Taiwan; 6 Department of Mathematics, Tamkang University, Taipei, Taiwan; 7 Department of Medical Imaging and Radiological Sciences, Kaohsiung Medical University, Kaohsiung, Taiwan; 8 Graduate Institute of Biomedical Electronics and Bioinformatics, National Taiwan University, Taipei, Taiwan; 9 Department of Radiology, Tri-Service General Hospital, National Defense Medical Center, Taipei, Taiwan; National Yang-Ming University, Taiwan

## Abstract

**Background:**

There is an emerging interest in using magnetic resonance imaging (MRI) T2* measurement for the evaluation of degenerative cartilage in osteoarthritis (OA). However, relatively few studies have addressed OA-related changes in adjacent knee structures. This study used MRI T2* measurement to investigate sequential changes in knee cartilage, meniscus, and subchondral bone marrow in a rat OA model induced by anterior cruciate ligament transection (ACLX).

**Materials and Methods:**

Eighteen male Sprague Dawley rats were randomly separated into three groups (*n* = 6 each group). Group 1 was the normal control group. Groups 2 and 3 received ACLX and sham-ACLX, respectively, of the right knee. T2* values were measured in the knee cartilage, the meniscus, and femoral subchondral bone marrow of all rats at 0, 4, 13, and 18 weeks after surgery.

**Results:**

Cartilage T2* values were significantly higher at 4, 13, and 18 weeks postoperatively in rats of the ACLX group than in rats of the control and sham groups (*p*<0.001). In the ACLX group (compared to the sham and control groups), T2* values increased significantly first in the posterior horn of the medial meniscus at 4 weeks (*p* = 0.001), then in the anterior horn of the medial meniscus at 13 weeks (*p*<0.001), and began to increase significantly in the femoral subchondral bone marrow at 13 weeks (*p* = 0.043).

**Conclusion:**

Quantitative MR T2* measurements of OA-related tissues are feasible. Sequential change in T2* over time in cartilage, meniscus, and subchondral bone marrow were documented. This information could be potentially useful for *in vivo* monitoring of disease progression.

## Introduction

Osteoarthritis (OA) is a common debilitating joint disease that primarily affects the elderly. It is characterized by the progressive degeneration of cartilage, causing chronic joint pain and physical disability. For the last decade, the use of magnetic resonance imaging (MRI) has greatly improved the understanding of the disease process underlying OA [1], [2]. Quantitative MRI measurements (such as contrast enhanced T1, T1ρ, and T2 relaxation time mapping) are capable of providing information on the matrix composition and macromolecular alterations in degenerating cartilage[3]–[6].

T2 mapping is indeed one of the most extensively studied MRI techniques in the evaluations of OA. Previous work demonstrated that the T2 value is sensitive to the integrity of the collagen network [7] and hydration status of cartilage [8], [9]. Increased T2 value has been observed consistently in both *in vivo* [10] and *ex vivo* [7] cartilage degeneration models. Recently, there has been considerable interest in using T2* as a proxy measurement for T2 in the evaluation of OA [11], [12]. T2* is the apparent transverse relaxation time which is dependent on the field inhomogeneity. It is related to T2 by the equation 1/T2* = 1/T2+1/(γΔB), where γ is gyromagnetic ratio and ΔB represents field inhomogeneity. Mamisch et al. demonstrated that both T2 and T2* values reflect similar changes in the OA articular cartilage [12]. Nonetheless, T2* (compared to T2) measurement has the advantage of decreased specific absorption rate and shorter image acquisition time, and hence offers the opportunity for three dimensional high resolution imaging. Further experiments may be needed to fully characterize T2* change in OA in order to better serve as an imaging biomarker.

With many MRI studies focused on the changes of cartilage in OA, few have examined the changes in adjacent structures (i.e., meniscus and subchondral bone marrow). Emerging evidence points to a role of these structures in the pathology of OA. For instance, subchondral bone marrow lesions have been associated with the presence of clinical symptoms [13], [14], progression of cartilage degeneration [15], and bone attrition [16]. In addition, meniscal damage has also been regarded as a potent risk factor for the onset and progression of knee OA [17]–[20]. Given their importance, gaining further insights into the dynamics of these structures during the progression of OA is warranted. Such information can potentially contribute to the early identification, diagnosis, and/or staging of the disease.

The objective of present study was to examine the changes in the cartilage, meniscus, and subchondral bone marrow in a rat model of OA induced by anterior cruciate ligament transection (ACLX), using magnetic resonance (MR) T2* measurement. In addition, end-point histology of the knee was performed to correlate histological changes with results of MR measurement.

## Materials and Methods

### Ethics Statement

This study was performed in strict accordance with the recommendations in the Guide for the Care and Use of Laboratory Animals of the National Institutes of Health. The protocol was approved by the Institutional Animal Care and Use Committee (IACUC) of the National Defense Medical Center (Permit Number: IACUC-06-103). All experiments were performed under isoflurane anesthesia, and every effort was made to minimize suffering.

### Animal Preparation

**Figure 1:**
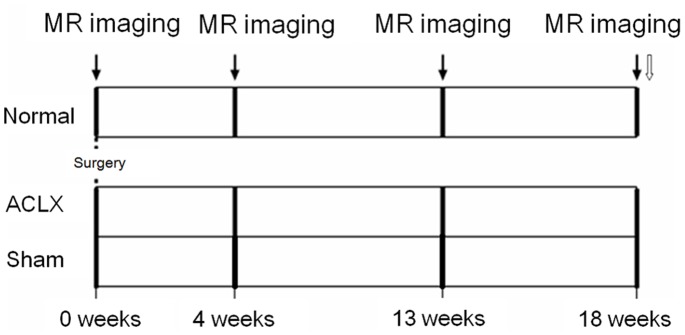
An overview of the protocol design in this study. (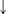) MR imaging; (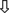) Histologic analysis.

Eighteen male Sprague Dawley rats aged 8 weeks and weighing around 300 g were randomly allocated into three groups (*n = *6 for each group). Rats (at week 0, right after grouping) received MR T2* imaging followed by either i) no intervention (group 1); ii) right ACLX, while the left anterior cruciate ligaments (ACLs) were left intact (group 2); or iii) sham surgery (the skin of the right knee was surgically wounded while the left knee was left intact (group 3). The experimental protocol is illustrated in Figure 1. Both knees of all rats were assessed by MR T2* measurement at week 4, 13, and 18.

### MR T_2_* Measurement

The rats were first anesthetized by inhalation of an isoflurane-oxygen mixture supplied via a tiny pipe directed to the rat’s nose and then positioned supinely with the forelegs fixed to the side and the rear legs stretched straight in a custom-made, MR-compatible device. A birdcage coil with an inner diameter of 72 mm was used as the transmitter coil, and a separate quadrature surface coil (Bruker, Ettlingen, Germany) was placed above both knee joints to achieve maximum signal reception. The entire device was placed in an Oxford Instruments (Bruker, Ettlingen, Germany) 200/300 magnet (4.7 T, 33 cm clear bore) equipped with an actively shielded Oxford gradient coil (16 cm inner diameter, 18 G/cm, 200 µs rise time).

After three-plane tripilot imaging, 20 contiguous axial T2-weighted images were acquired for the purpose of later slice positioning using a turbo spin echo sequence with repetition time (TR) = 3500 ms, echo time (TE) = 40 ms, echo train length = 8, slice thickness (SLTH) = 0.5 mm, matrix size = 256×128, in-plane resolution = 156×312 µm^2^, number of excitations (NEX) = 4, bandwidth = 50.0 kHz, and acquisition time = 3 min 44 s. To measure T2* relaxation times of OA-related tissues, a fast gradient echo (GRE) sequence was performed to reduce the minimal TE. In this experiment, 7 slices (i.e., two sets of three contiguous slices covering most of the femorotibial cartilage plus one sagittal plane placed in the middle of each knee joint) were acquired for one knee joint of each rat. Following the slice positioning, T2* measurements were made using a multislice multiecho fast gradient echo sequence with TR = 400 ms, TE = 3.5, 8.5, 13.5, 18.5, 23.5, 28.5, 33.5, and 38.5 ms, SLTH = 0.65 mm, matrix size = 256×256, in-plane resolution = 117×100 µm^2^, NEX = 18, flip angle = 30°, bandwidth = 69.4 kHz, and acquisition time = 30 min 43 s. The total scan time including the rat preparation was about 1.5 hours. Although GRE sequence is prone to susceptibility artifact, the use of small voxel size, increased bandwidth, and higher echo-train length [21] in the present study were beneficial in minimizing this effect.

### Image Processing

#### T2* calculation

**Figure 2:**
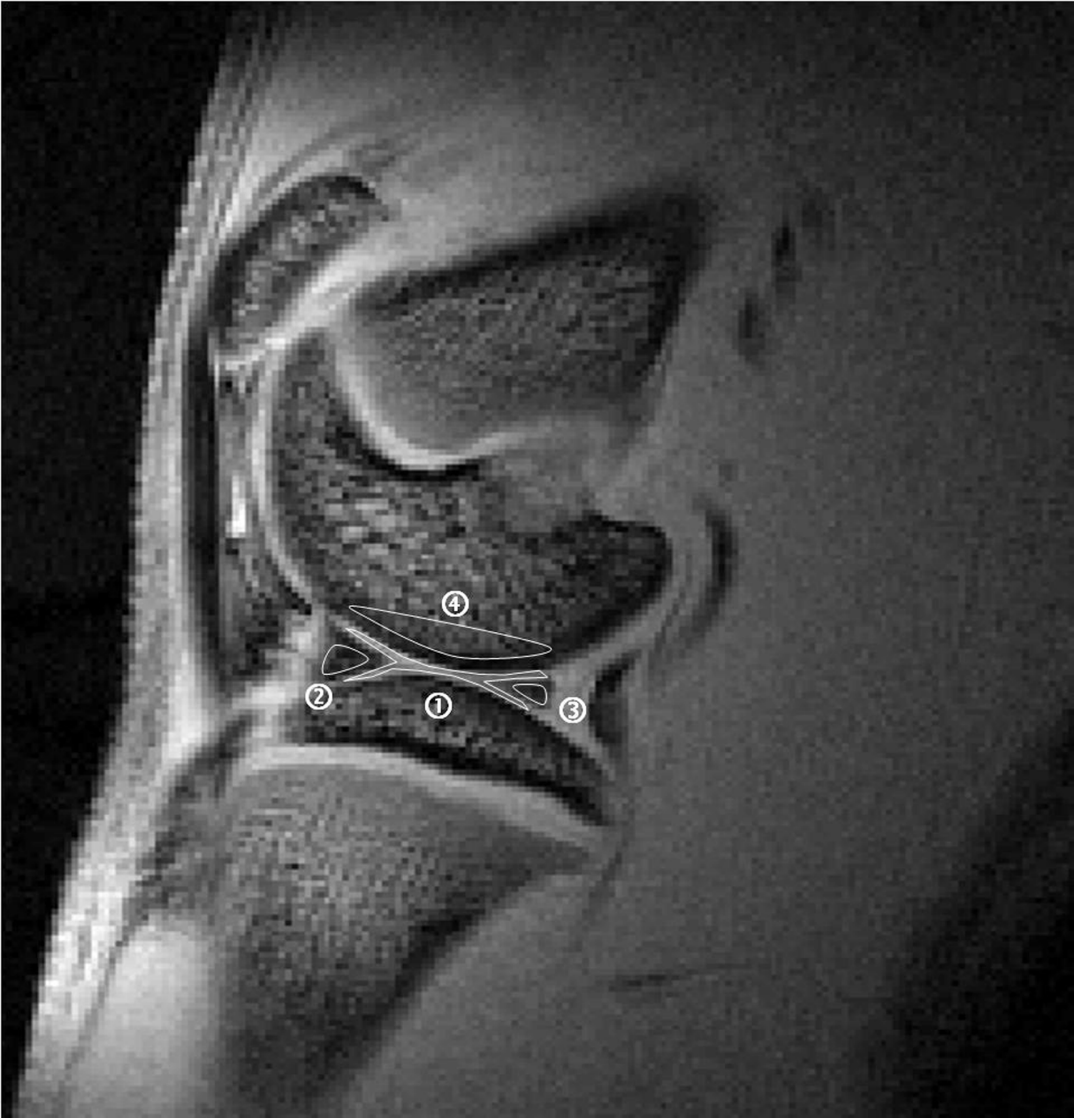
ROIs selected for T2* calculation using the first-echo image. 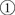
 articular cartilage 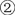 anterior horn of the medial meniscus 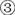 posterior horn of the medial meniscus, and 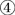 femoral subchondral bone marrow.

After the image acquisition was completed, all data were transferred to a stand-alone personal computer. To calculate the mean values of the signal intensity, regions of interest (ROIs) were drawn manually on the femorotibial cartilage, anterior and posterior horn of the medial menisci, and femoral subchondral bone marrow by referencing to the first-echo image (Figure 2). To reduce the partial volume effect, the borders of the articular cartilage and menisci were not included in the ROIs. In addition, the selected ROI of the femoral subchondral bone marrow only consisted of the area thought to be most affected by cartilage degradation (i.e., extending from below the anterior margin of the anterior horn of the medial meniscus to the posterior margin of the posterior horn of the medial meniscus) [22]. The average numbers of pixels included in the ROIs of knee cartilage, meniscus and subchondral bone marrow were approximately 415, 143, 523 respectively. To minimize manual discrepancies in the positioning of the ROIs, the ROIs were drawn by two operators well trained in knee cartilage MR imaging (PHT, MCC) and were reconfirmed by an experienced musculoskeletal radiologist (GSH). Results shown in this manuscript are the mean of two measurements.

Due to the advantage of reducing fitting error in low signal-to-noise MR images using regional-based method [23], the T2* relaxation time of the tissues was calculated zone by zone in the selected ROIs using the least-square, single-exponential curve-fitting method on a MATLAB 7.0 (MathWorks, Natick, MA, USA) software platform. Two parameters, spin density (M_0_) and apparent transverse relaxation time (T2*), were determined by fitting the signal magnitude from echoes of the multislice multiecho experiment to a monoexponential decay model. Because of the relatively short T2* relaxation time of the menisci and subchondral bone marrow, only the first 4 TE data were used in the T2* fitting for all knee structures.

### Histological Analysis

The rats were sacrificed following imaging at week 18. Both knee joints were removed, fixed in neutral formalin, and decalcified in a rapid decalcifier (Nihon Shiyaku Industries Ltd., Osaka, Japan). After decalcification, the knee joint tissues were cut in half along the midsagittal line. Four samples of each joint were taken (one each from the medial and lateral femoral condyles, and from the lateral and medial tibial plateau), paraffin-embedded, and cut into 5-µm sections (8 sections for hematoxylin and eosin and 8 sections for Safranin O staining). Joint health (cartilage health) was graded in all specimens using Mankin scores as previously described [24]. In addition, the specimens of the anterior and posterior horn of the medial menisci and femoral subchondral bone marrow were examined.

### Statistical Analysis

The mean value and standard deviation (SD) of the T2* values for the OA-related tissues were first calculated in each group. To compare differences in the longitudinal effects of T2* values among groups due to their dependence on repeated measurement, Generalized Estimating Equations (GEE) multiple linear regression [25] was used to assess the interaction of groups (ACLX, Control, and Sham) or side (Right and Left knees) with time (0, 4, 13, and 18 weeks). SPSS v19.0 software (SPSS Inc., Chicago, IL, USA) was used to analyze the results of GEE with autoregressive correlation. The Mann-Whitney *U* test was used to compare Mankin scores between groups. The interobserver variability of the T2* measurements was assessed by calculating the Pearson correlation coefficient. The root-mean-square average coefficient of variation (CV_RMS_) was used to assess the reproducibility of the T2* measurements, and CV_RMS_ values <10% were interpreted as good. *P* values <0.05 were regarded as statistically significant.

## Results

### MR T2* Analysis

#### Cartilage

**Figure 3:**
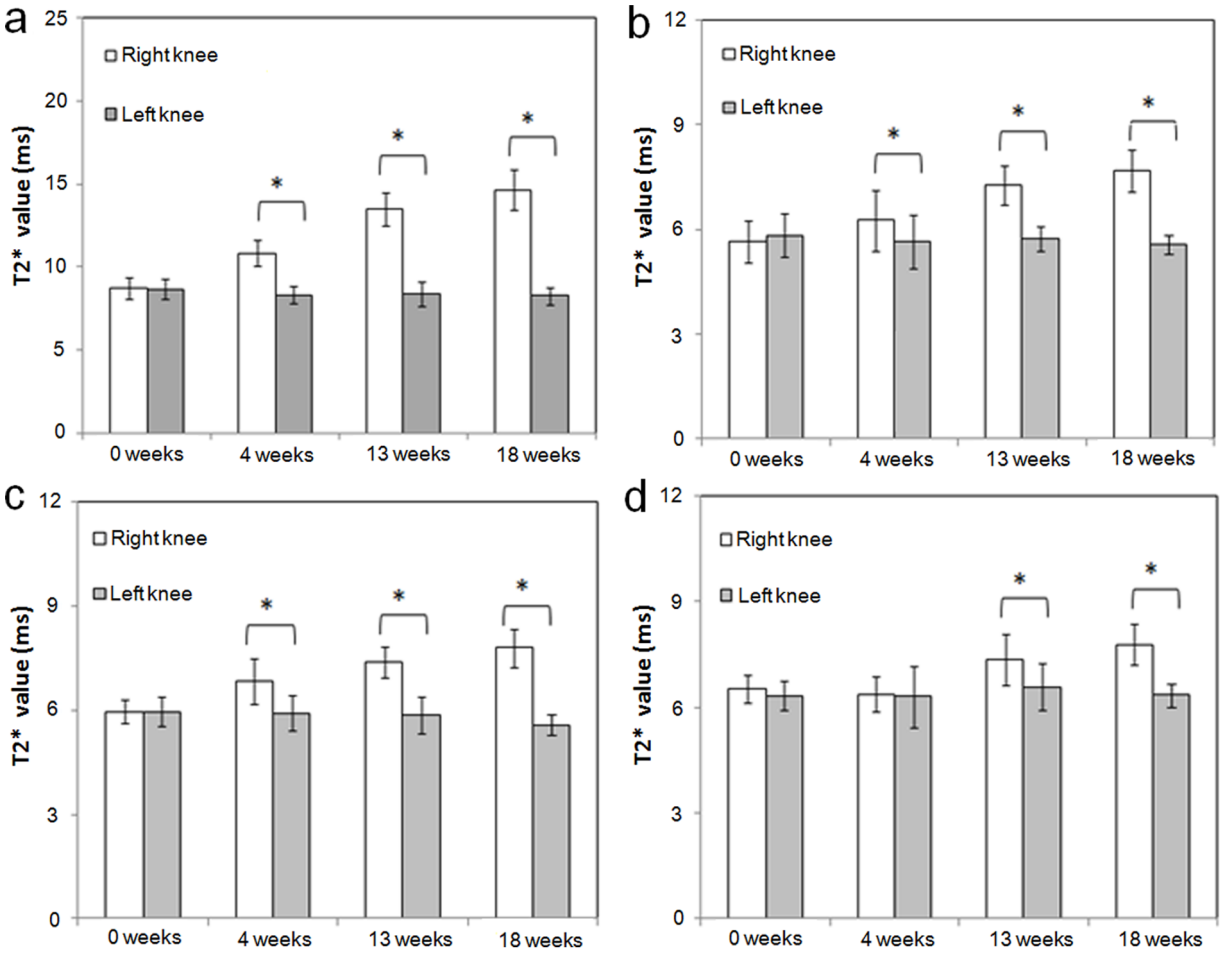
Plots of the T2* values (mean ± SD) for the ACLX group. Values are for cartilage (a), anterior horn of the medial meniscus (b), posterior horn of the medial meniscus (c), and femoral subchondral bone marrow (d) at weeks 0, 4, 13, and 18, separately measured in the operated right knees and the intact left knees of all rats. Asterisks indicate significant differences (*p*<0.05).

**Figure 4:**
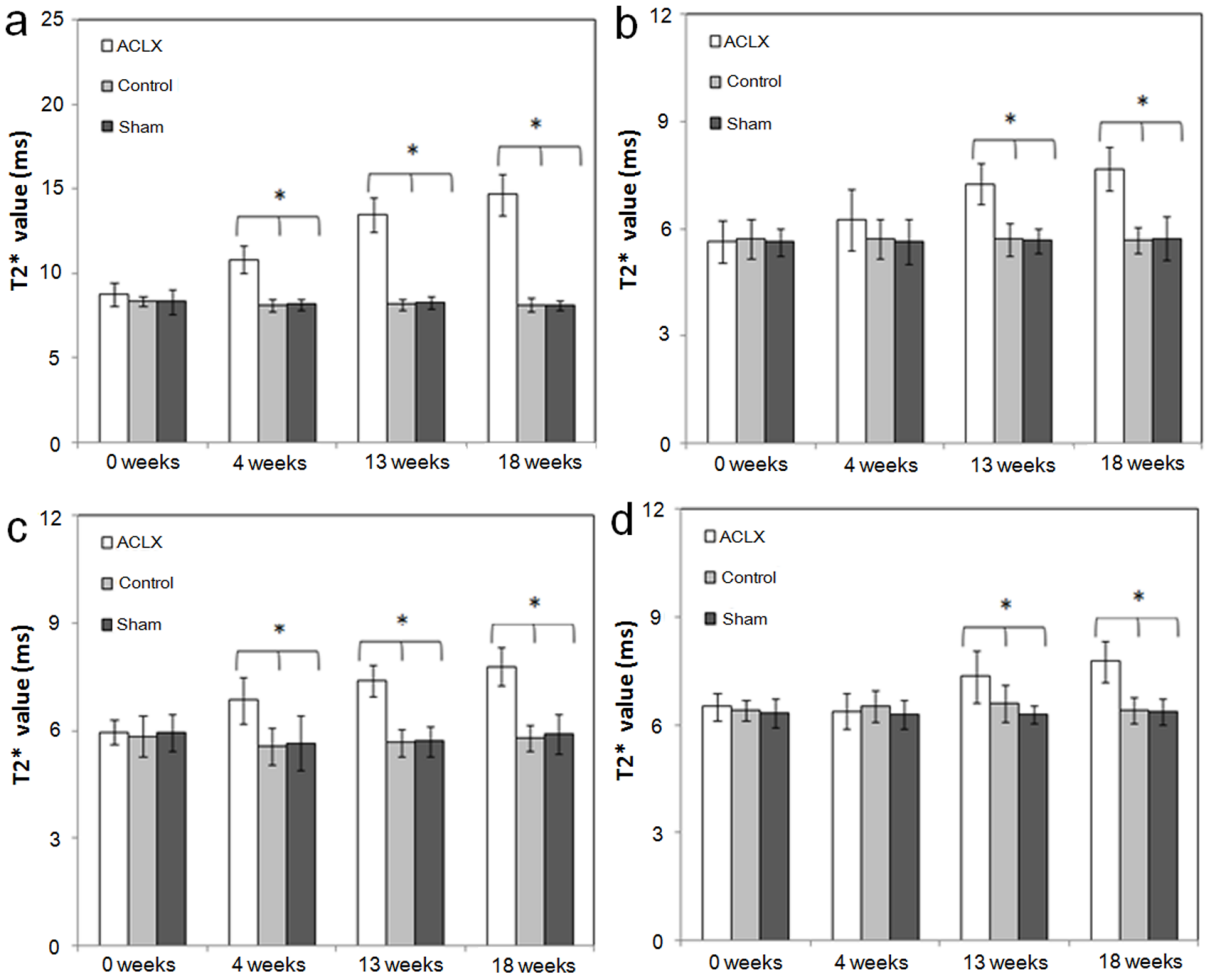
Plots of the T2* values (mean ± SD) in the right knee joints of three groups. The values are for cartilage (a), anterior horn of the medial meniscus (b), posterior horn of the medial meniscus (c), and femoral subchondral bone marrow (d) at weeks 0, 4, 13, and 18. Asterisks indicate significant differences (*p*<0.05).

Results of MR T2* measurements in the ACLX group are shown in Figure 3 and Table 1. As shown in Table 1, the initial T2* values (before ACLX) were not significantly different between the right and left knees for cartilage (*p*-value = 0.521). In addition, the longitudinal effect for the intact left knees was not significant. The changes in T2* value at weeks 4, 13, and 18 (compared to week 0) were all significantly higher in the right knees of the ACLX group (on average, 2.397, 4.971, and 6.289 ms, respectively; all *p*-values <0.001) than in the left knees (Table 1). In addition, the among-group difference in the initial cartilage T2* value before ACLX (week 0) was not significantly different (Table 2). The change in T2* value at 4, 13, and 18 weeks (compared to week 0) were all significantly higher for operated knees in the ACLX group than knees in the control group (on average, 2.295, 4.914, and 6.123 ms. respectively; all *p*-values <0.001). There was no statistically significant difference between the control and sham groups in the change in T2* value at weeks 4, 13, and 18 (*p*-values = 0.794, 0.576, and 0.859, respectively; Figure 4).

**Table 1:** Comparisons of four T2* values between right (Side 1) and left knees (Side 0) over 18 weeks in the ACLX group using multiple linear regression with generalized estimating equations.

Parameter	B	Std. error	Wald chi-square	*p*-value
**Cartilage**				
Side^a^ (Right vs. Left)	0.092	0.1427	0.412	0.521
Week 18 vs. Week 0	–0.400	0.1925	4.310	0.038
Week 13 vs. Week 0	–0.263	0.2085	1.593	0.207
Week 4 vs. Week 0	–0.340	0.2291	2.199	0.138
Side* Week 18	6.289	0.2800	504.597	<0.001
Side* Week 13	4.971	0.3558	195.238	<0.001
Side* Week 4	2.397	0.2438	96.623	<0.001
**Anterior meniscus**				
Side (Right vs. Left)	–0.196	0.2437	0.645	0.422
Week 18 vs. Week 0	–0.267	0.1882	2.018	0.155
Week 13 vs. Week 0	–0.101	0.2062	0.241	0.624
Week 4 vs. Week 0	–0.186	0.1897	0.961	0.327
Side* Week 18	2.315	0.2997	59.666	<0.001
Side* Week 13	1.730	0.3137	30.409	<0.001
Side* Week 4	0.807	0.3310	5.936	0.015
**Posterior meniscus**				
Side (Right vs. Left)	–0.005	0.1214	0.002	0.966
Week 18 vs. Week 0	–0.387	0.1701	5.184	0.023
Week 13 vs. Week 0	–0.107	0.1439	0.556	0.456
Week 4 vs. Week 0	–0.043	0.1646	0.068	0.794
Side* Week 18	2.219	0.2418	84.204	<0.001
Side* Week 13	1.536	0.1719	79.809	<0.001
Side* Week 4	0.934	0.2755	11.489	0.001
**Subchondral bone marrow**				
Side (Right vs. Left)	0.181	0.1452	1.556	0.212
Week 18 vs. Week 0	–0.001	0.1385	<0.001	0.992
Week 13 vs. Week 0	0.242	0.2403	1.011	0.315
Week 4 vs. Week 0	–0.043	0.2613	0.027	0.869
Side* Week 18	1.253	0.2345	28.535	<0.001
Side* Week 13	0.586	0.2882	4.131	0.042
Side* Week 4	–0.105	0.2677	0.154	0.695

a Side 1 = Right knee and Side 0 = Left knee (the reference knee).

* The interaction between two variables in the models.

B: regression coefficient.

**Table 2:** Comparisons of cartilage T2* values among three groups over 18 weeks using multiple linear regression with generalized estimating equations.

Cartilage Parameter	B	Std. error	Waldchi-square	*p*-value
(Intercept)	8.383	0.0754	12369.934	<0.001
Group 3^a^ (Sham vs. Control)	–0.052	0.2215	0.055	0.815
Group 2^a^ (ACLX vs. Control)	0.381	0.1946	3.844	0.050
Week 18 vs. Week 0	–0.233	0.1572	2.202	0.138
Week 13 vs. Week 0	–0.207	0.0920	5.044	0.025
Week 4 vs. Week 0	–0.238	0.1119	4.521	0.033
Group 3* Week 18	0.053	0.2994	0.032	0.859
Group 3* Week 13	0.160	0.2855	0.312	0.576
Group 3* Week 4	0.068	0.2613	0.068	0.794
Group 2* Week 18	6.123	0.3548	297.776	<0.001
Group 2* Week 13	4.914	0.3764	170.426	<0.001
Group 2* Week 4	2.295	0.2040	126.565	<0.001

a Group 1 = Control group; Group 2 = ACLX group; Group 3 = Sham group.

* The interaction between two variables in the models.

B: regression coefficient.

#### Anterior horn of the medial meniscus

The initial T2* values (before ACLX) were not significantly different between the right and left knees for the anterior horn of medial meniscus (*p*-value = 0.422). In addition, the longitudinal effect for the intact left knees was not significant. The changes in T2* value at weeks 4, 13, and 18 (compared to week 0) were significantly higher in the right knees than in the left knees (on average, 0.807, 1.730, and 2.315 ms, respectively; *p*-values 0.015, <0.001, and <0.001, respectively; Table 1 and Figure 3b). Moreover, as shown in Table 3, the changes in T2* value at 13 and 18 weeks after ACLX were significantly higher in the right knees of the ACLX group than in the right knees of the control group (1.622 and 2.062 ms, respectively, *p-*value <0.001). There were no significant differences in the changes in T2* value at weeks 4, 13, and 18 between the control and sham groups (*p*-values = 0.961, 0.879, and 0.607, respectively; Figure 4b).

**Table 3:** Comparisons of T2* values in the anterior meniscus among three groups over 18 weeks using multiple linear regression with generalized estimating equations.

Anterior meniscus Parameter	B	Std. error	Waldchi-square	*p*-value
(Intercept)	5.708	0.1531	1390.093	<0.001
Group 3^a^ (Sham vs. Control)	–0.083	0.1860	0.198	0.656
Group 2^a^ (ACLX vs. Control)	–0.075	0.2229	0.114	0.735
Week 18 vs. Week 0	–0.014	0.1934	0.006	0.941
Week 13 vs. Week 0	0.006	0.2269	0.001	0.977
Week 4 vs. Week 0	0.011	0.2479	0.002	0.963
Group 3* Week 18	0.134	0.2600	0.265	0.607
Group 3* Week 13	0.040	0.2644	0.023	0.879
Group 3* Week 4	0.016	0.3356	0.002	0.961
Group 2* Week 18	2.062	0.2875	51.456	<0.001
Group 2* Week 13	1.622	0.3286	24.364	<0.001
Group 2* Week 4	0.609	0.3640	2.801	0.094

a Group 1 = Control group; Group 2 = ACLX group; Group 3 = Sham group.

* The interaction between two variables in the models.

B: regression coefficient.

#### Posterior horn of the medial meniscus

The initial T2* values (before ACLX) were not significantly different between the right and left knees for the posterior horn of medial meniscus (*p*-value = 0.966). In addition, the longitudinal effect for the intact left knees was not significant. The changes in T2* value at week 4, 13 and 18 after ACLX were all significantly higher in the right knees than in the left knees (on average, 0.934, 1.536, and 2.219 ms respectively, *p*-values = 0.001, <0.001, and <0.001, respectively; Table 1 and Figure 3c). Moreover, the change in T2* values at 4, 13, and 18 weeks after ACLX were all significantly higher in the right knees of the ACLX group than in the right knees of the control group (1.158, 1.595, and 1.884 ms, respectively, all *p*-values ≦0.001; Table 4) and not significantly different between the normal control and sham groups (*p*-values = 0.947, 0.829, and 0.929, respectively; Figure 4c).

**Table 4:** Comparisons of T2* values in the posterior meniscus among three groups over 18 weeks using multiple linear regression with generalized estimating equations.

Posterior meniscus Parameter	B	Std. error	Waldchi-square	*p*-value
(Intercept)	5.848	0.1580	1369.247	<0.001
Group 3^a^ (Sham vs. Control)	0.105	0.2117	0.244	0.621
Group 2^a^ (ACLX vs. Control)	0.111	0.1822	0.372	0.542
Week 18 vs. Week 0	–0.052	0.1801	0.084	0.772
Week 13 vs. Week 0	–0.167	0.2314	0.518	0.472
Week 4 vs. Week 0	–0.268	0.2468	1.176	0.278
Group 3* Week 18	0.022	0.2489	0.008	0.929
Group 3* Week 13	–0.068	0.3147	0.047	0.829
Group 3* Week 4	–0.024	0.3617	0.004	0.947
Group 2* Week 18	1.884	0.2660	50.167	<0.001
Group 2* Week 13	1.595	0.2580	38.201	<0.001
Group 2* Week 4	1.158	0.3359	11.895	0.001

a Group 1 = Control group; Group 2 = ACLX group; Group 3 = Sham group.

* The interaction between two variables in the models.

B: regression coefficient.

#### Femoral subchondral bone marrow

The initial T2* values (before ACLX) were not significantly different between the right and left knees for the subchondral bone marrow (*p*-value = 0.212). The changes in T2* value were not significantly different between the right knees and left knees at week 4 (*p-*value = 0.695) but at weeks 13 and 18, they were significantly higher in the right knee (0.586 and 1.253 ms, respectively) than in the left knee (*p*-values = 0.042 and <0.001, respectively; Table 1 and Figure 3d); significantly higher in the right knees of the ACLX group (0.627 and 1.253 ms, respectively) than in the right knees of the control group (*p*-values = 0.043 and <0.001, respectively; Table 5), but not significantly different between the control and sham groups (*p*-values = 0.550, 0.243, and 0.825, respectively, at 4, 13, and 18 weeks; Figure 4d).

**Table 5:** Comparisons of T2* values in femoral subchondral bone marrow among three groups over 18 weeks using multiple linear regression with generalized estimating equations.

Subchondral bone marrow Parameter	B	Std. error	Wald chi-square	*p*-value
(Intercept)	6.406	0.0812	6218.056	<0.001
Group 3^a^ (Sham vs. Control)	–0.064	0.1372	0.220	0.639
Group 2^a^ (ACLX vs. Control)	0.108	0.1327	0.666	0.414
Week 18 vs. Week 0	–0.001	0.1380	0.000	0.993
Week 13 vs. Week 0	0.201	0.1539	1.699	0.192
Week 4 vs. Week 0	0.101	0.1262	0.637	0.425
Group 3* Week 18	0.043	0.1971	0.049	0.825
Group 3* Week 13	–0.255	0.2182	1.364	0.243
Group 3* Week 4	–0.128	0.2150	0.356	0.550
Group 2* Week 18	1.253	0.2574	23.687	<0.001
Group 2* Week 13	0.627	0.3093	4.106	0.043
Group 2* Week 4	–0.249	0.1876	1.759	0.185

a Group 1 = Control group; Group 2 = ACLX group; Group 3 = Sham group.

* The interaction between two variables in the models.

B: regression coefficient.

In the normal and sham-control groups, the T2* value remained nearly constant over time and did not differ significantly between the right and left knee (*p*>0.1). The interobserver correlation coefficients for the ROI measurements were high (r = 0.93, *p*<0.01) in all rats. The CV_RMS_ of the T2* measurements in the normal rat knee cartilage, menisci, and femoral subchondral bone marrow were 6.87%, 7.58%, and 7.66%, respectively, indicating good reproducibility.

### Histologic Analysis

**Figure 5:**
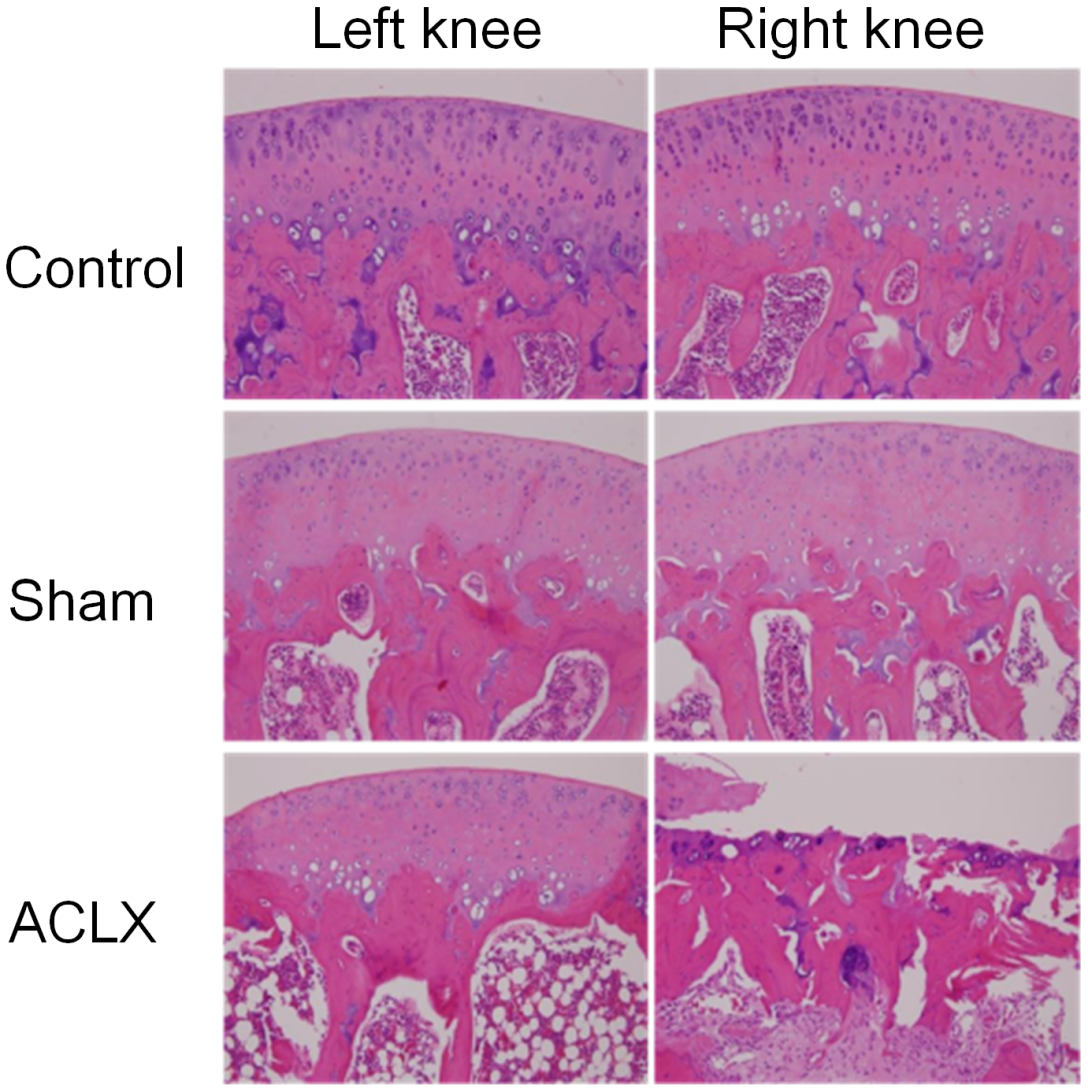
Histologic confirmation of changes in the articular cartilage and femoral subchondral bone marrow. In the ACLX group, severe cartilage degeneration including loss of chondrocytes, extracellular matrix deterioration, and fibrovascular proliferation of the subchondral bone marrow are seen in the operated right knee. In contrast, the cartilage and subchondral bone of the femorotibial joint in the other knee joint have a normal histologic appearance.

**Figure 6:**
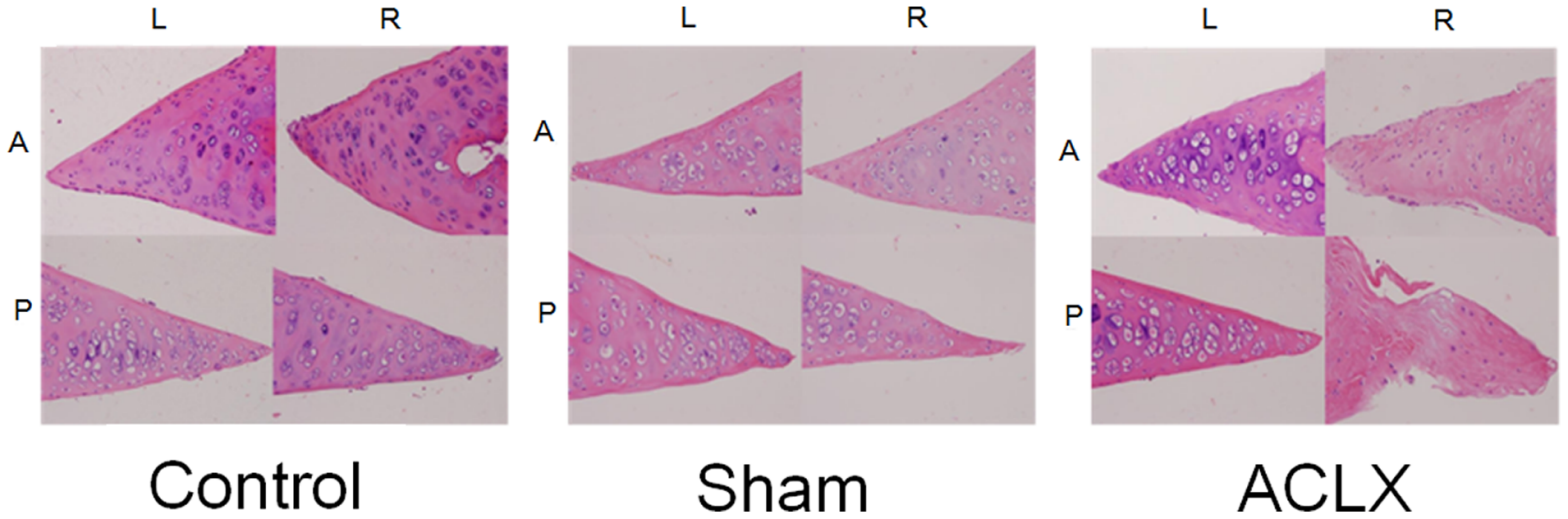
Histologic confirmation of changes in the anterior and posterior horn of the medial menisci. In the ACLX group, severe degeneration of the posterior horn of the medial meniscus including decreased cellularity, markedly degenerated cartilage matrix with myxoid changes, and fibrillation of the surface are seen in the operated right knee. In contrast, only relatively mild degenerative change is seen in the anterior horn of the medial meniscus. Moreover, no significant histologic changes are seen in the menisci of the control and sham groups.

Histologic examination of the cartilages in the right femorotibial joints of ACLX rats showed erosive defects, exposure of the femoral subchondral bone plate, and fibrovascular change of the femoral subchondral bone marrow at the study endpoint (Figure 5). In the control and sham groups, the cartilage, femoral subchondral bone marrow of the femorotibial joint, and anterior and posterior horns of medial meniscus had normal appearance. In the ACLX rats, the posterior horn of the medial menisicus showed decreased cellularity, markedly degenerative matrix with myxoid change, and fibrillation of the surface at the study endpoint (Figure 6), and the anterior horn of medial meniscus of the right knee showed mild degenerative changes in the matrix. The Mankin scores at week 18 for the right knee differed significantly between the ACLX group (9.8±0.9 [right knee] and 1.3±0.8 [left knee]) and the control (0.6±0.5 [right knee] and 0.8±0.7 [left knee]) and sham (1.0±0.8 [right knee] and 0.8±0.7 [left knee]; *p*<0.005) groups.

## Discussion

Our study demonstrated the feasibility of using MR T2* as an imaging biomarker to monitor the sequential changes in articular cartilage and its adjacent structures in a rodent model of knee OA. We noted a tendency towards increased T2* of the articular cartilage, meniscus and subchondral bone marrow during progression of the disease. Histologic examination confirmed the presence of corresponding pathologic changes in the above mentioned areas. Since most existing data on cartilage T2* values are derived from studies on human subjects or large animal subjects using clinical scanners, a direct comparison of our results with those in the previous published literature is inappropriate. T2 relaxation time is sensitive to the interactions between water molecules and macromolecular concentration, as well as the structure of the extracellular matrix [26], [27]. The increase in the T2* of the degenerated cartilage and meniscus as observed in the present study is probably the result of the characteristic collagen loss and increased water content within the structures [28], whereas the increase in T2* of the subchondral bone marrow can be attributed to “bone marrow edema” lesions, which are hyperintense on T2-weighted images. Zanetti et al. found that, at histologic examination, specimens with bone marrow edema pattern on imaging consisted of mainly normal bone marrow, with some areas of bone marrow necrosis, abnormal trabeculae, and fibrotic changes [29]. Histologic examination of subchondral bone marrow in present study mainly showed normal or fibrovascular change, which is in agreement with previous study on human subjects [29]. Notably, movement of synovial fluid into degenerated cartilage because of the presence cartilage defects may falsely increase the measured T2*. Fluid-attenuated inversion recovery (FLAIR) is a feasible technique which can eliminate the synovial fluid signal, but at the expense of lower signal-to-noise ratio and longer image acquisition time. In present study, whenever presence of synovial fluid was noted in the cartilage defects and tibiofemoral joint space, we placed two separated ROIs on the femoral and tibial cartilage. In this way, the ROIs defined in this study specifically excluded voxels representing cartilage defects filled by synovial fluid, minimizing the aforementioned effect.

ACL is a crucial part of knee complex which provides mechanical stability to the joint. Injury to the ACL leads to change in kinematics, causing increased loading in the cartilage area which is not conditioned to frequent load bearing. Hypothetically, this causes destruction in the collagen network of the articular cartilage and hence initiates OA. Previous reports have consistently found associated medial meniscus damage after ACLX [30], [31]. In the present study, an almost simultaneous increase in T2* values of cartilage and meniscus was noted at week 4 after ACLX. Although the cartilage and meniscal T2* changes before week 4 were not captured by the current experimental protocol, this observation indicating association rather than proving causation may also point to the possibility that injuries of the cartilage and meniscus after ACLX are both consequences of knee instability. Previous studies on canine ACLX supported a similar notion. Gross meniscal tearing did not always occur before cartilage ulceration developed, and the relative severity of cartilage damage was not correlated with the severity of meniscus damage [31]. We also found that T2* values of the posterior horn of the medial meniscus differed significantly between the operated and control knees beginning week 4 after ACLX, and that these differences occurred before changes in the anterior horn. On the other hand, the degeneration on histological examination was more severe in the posterior horn of medial meniscus than in the anterior horn, which suggests that damage to the anterior horn was relatively mild in rat knees after ACLX.

Subchondral bone edema is not an uncommon lesion identified in advanced OA. Previous studies have related this change to the presence of joint pain [13], [14]. We found that increased in T2* values of subchondral bone marrow occurred in the later stage of disease, following changes in cartilage and menisci. This may suggest that subchondral bone marrow edema is triggered by cartilage and/or meniscal degeneration.

The ACLX model has been widely used experimentally to study OA. It has been successfully applied in dogs, pigs, and rats, with numerous publications describing the pathogenesis, molecular events, and effects of OA using this model [31]–[34]. Alternatively, surgical destabilization of the medial meniscus represents another animal model that induces a less severe form of OA in the experimental subjects [35]. However, destruction of subchondral bone structures is rarely observed in this latter model. The ACLX model has been combined with partial medial meniscectomy to accelerate the progression of OA [33], [36]. Nevertheless, for consistency with our previous studies, we used the ACLX model in present study [37], [38].

The highly organized collagen network of the knee cartilage results in the orientation-dependent dipolar interactions. The increase in T2* values as the fibrils in the cartilage approach an angle of approximately 55° relative to the main magnetic field is known as the magic angle effect [39]. This effect was found to be minor in recent studies[40]–[42]. Nonetheless, to mitigate the influence of magic angle effect, curved articular surfaces were avoided, with only central cartilage located within weight-bearing areas being selected for analysis in present study.

Several limitations in present study should be noted. One important limitation is that the very short T2* component of knee structures cannot be assessed, as the first TE of multiecho gradient echo sequence was at 3.5 ms. Williams et al. have demonstrated the feasibility of using ultra-short TE (UTE) (with TE = ∼0.5 ms or shorter) to probe the very short T2* components of cartilage [43]. In addition, the same group also showed that T2* mapping of the meniscus using the UTE technique can reveal meniscal injury with high sensitivity [44]. Furthermore, with the multi-component T2* fitting on the signal decay curve acquired with UTE sequences, contributions from short T2* tissue can be isolated and identified [45]. These methodologies are promising future approaches for detecting subtle changes due to OA. Other important limitations of the study include the relatively small sample size and potential contamination by partial volume effects owing to the small size of knee structures. Higher resolution using three dimensional MRI sequences to better resolve the knee structures could potentially reduce the partial volume effects.

## Conclusion

In summary, our study demonstrated the feasibility of using MR T2* to monitor the sequential changes in articular cartilage and its adjacent structures in the rat ACLX model of OA. We documented the sequential T2* changes in the cartilage, meniscus, and subchondral bone marrow during the progression of OA and provided histologic correlation at the end point. This *in vivo* model can potentially serve as a reliable platform to monitor OA disease progression and to evaluate therapeutic efficacy of candidate agents.
